# Emerging viral zoonotic diseases: time to address the root causes

**DOI:** 10.1186/s42269-023-00993-3

**Published:** 2023-02-02

**Authors:** Usman Abubakar Haruna, Ahmad Dalhatu Muhammad, Don Eliseo Lucero-Prisno

**Affiliations:** 1 Department of Biomedical Sciences, Nazarbayev University School of Medicine, Astana, Kazakhstan; 2 Faculty of Pharmaceutical Sciences, Ahmadu Bello University, Zaria, Nigeria; 3 Department of Global Health and Development, London School of Hygiene and Tropical Medicine, London, UK

To the Editor,

There have been multiple outbreaks of emerging infections since the turn of the century. A bulk of these diseases is caused by animals, including the Zika virus, Middle East Respiratory Syndrome (MERS), Ebola Virus Disease (EVD), Coronavirus Disease 2019 (COVID-19), Monkeypox (MPOX), and Adenovirus among others (Manirambona et al. 2022; Alarcon-Valdes et al. 2022). These diseases were formerly known to infect animals, but they now affect humans. We think that human activities such as land use and livestock practices, and the impact of climate change, are major contributing factors to the emergence and spread of viral zoonotic diseases. By addressing these root causes, we can reduce the risk of future outbreaks and improve public health.

The first human case of COVID-19 was reported in Wuhan, China, in December 2019. Subsequently, the virus spread around the world, resulting in over 650 million confirmed COVID-19 cases and nearly 6.6 million deaths (WHO 2022). On March 20, 2020, the World Health Organization (WHO) declared it a global pandemic. This disease also complicates the health of people with comorbid conditions and disrupts socioeconomic activities, resulting in economic contractions and the loss of jobs and other income sources. While the COVID-19 pandemic was still wreaking havoc, another outbreak of the monkeypox virus emerged and spread uncontrollably. According to the Centers for Disease Control and Prevention, there were about 83,434 confirmed cases and 110 fatalities globally as of December 22, 2022. Surprisingly, a majority of cases are in locations where the disease has never been an epidemic. Likewise, deaths as a result of monkeypox have occurred in non-endemic regions (CDC 2022). As a result, on July 23, 2022, the WHO declared it a Public Health Emergency of International Concern (PHEIC).

On July 17, 2022, Ghana declared an epidemic of Langya henipavirus (LayV), a zoonotic disease of public health concern related to the Nipah and Hendra viruses, as well as the Marburg virus disease. Marburg virus disease is related to EVD. The 2013–2016 EVD outbreak was unprecedented in recorded history, with cases primarily in Guinea, Liberia, and Sierra Leone (WHO 2014). While these viruses are highly unpredictable and have high epidemic potential, there is the absence of specific treatments and vaccines to help contain them. Disturbingly, they have a high case-fatality ratio, aside from the burden they exert on health systems (WHO 2014). Therefore, as these diseases continue to transcend borders and previously regional diseases become global, it is crucial to address the drivers of the spillover of these diseases.

Closer contact between humans and animals due to rapid urbanization and the destruction of natural habitats, wildlife hunting, livestock operations, climate change, animal domestication, and changing ecosystems are some factors that precipitate the emergence of these diseases (Alimi et al. 2021). Accordingly, there is a need to advance the conservation of tropical forests, regulate wildlife trade, and improve biosecurity around wildlife and livestock farms. These strategies are cost-effective and promote biodiversity (Alimi et al. 2021). As humans have no pronounced natural immunity against these viral zoonotic diseases, pandemics should not be our fate. The significance of these emerging viral zoonotic diseases, which brought about a public health emergency, underscores the requirement for concerted action across sectors to preserve health, biodiversity, and animal ecology. By working together and considering the interconnectedness of human, animal, and environmental health, we can more effectively prevent and control these diseases. Therefore, an integrated One Health strategy is essential for the prediction, prevention, detection, and control of diseases that are leaping from animals to humans.

However, research is needed to understand the complex factors that contribute to the emergence and spread of these diseases, including the role of human activities and the impact of climate change. These would help to unravel the full impact of these diseases on global health, including the economic and social costs, as well as the potential long-term consequences for public health. More research could focus on identifying and evaluating interventions that are effective at addressing the root causes of emerging viral zoonotic diseases. This could include the development of new technologies or practices to reduce the risk of disease emergence, as well as the evaluation of existing interventions to determine their effectiveness including the development of new tools and technologies for surveillance and the implementation of effective response strategies. By addressing these research questions, we can gain a deeper understanding of emerging viral zoonotic diseases and develop effective strategies to permanently address the root causes of this global health threat.

## Data Availability

Not applicable.
